# Non-Isothermal Oxidation Behavior and Mechanism of a High Temperature Near-α Titanium Alloy

**DOI:** 10.3390/ma11112141

**Published:** 2018-10-30

**Authors:** Peixuan Ouyang, Guangbao Mi, Peijie Li, Liangju He, Jingxia Cao, Xu Huang

**Affiliations:** 1 National Center of Novel Materials for International Research, Tsinghua University, Beijing 100084, China; oypx12@mails.tsinghua.edu.cn (P.O.); lipj@mail.tsinghua.edu.cn (P.L.); helj@mail.tsinghua.edu.cn (L.H.); 2 State Key Laboratory of Tribology, Department of Mechanical Engineering, Tsinghua University, Beijing 100084, China; 3 Aviation Key Laboratory of Science and Technology on Advanced Titanium Alloys, AECC Beijing Institute of Aeronautical Materials, Beijing 100095, China; caojingxia@sina.com (J.C.); huangxu621@163.com (X.H.); 4 Beijing Engineering Research Center of Graphene and Application, Beijing 100095, China

**Keywords:** titanium alloys, non-isothermal, oxidation, mechanism, lattice transformation, alloying elements

## Abstract

Non-isothermal oxidation is one of the important issues related to the safe application of high-temperature titanium alloys, so this study focuses on the non-isothermal oxidation behavior and mechanism of near-α titanium alloys. The thermogravimetry-differential scanning calorimetry (TGA/DSC) method was used to study the non-isothermal oxidation behavior of TA29 titanium alloy heated from room temperature to 1450 °C at a heating rate of 40 °C/min under pure oxygen atmosphere. The results show that non-isothermal oxidation behavior can be divided into five stages, including no oxidation, slow oxidation, accelerated oxidation, severe oxidation and deceleration oxidation; for the three-layer TiO_2_ scale, Zr, Nb, Ta are enriched in the intermediate layer, while Al is rich in the inner layer and Sn is segregated at the oxide-substrate interface, which is related to their diffusion rates in the subsurface α case. The oxidation mechanism for each stage is: oxygen barrier effect of a thin compact oxide film; oxygen dissolution; lattice transformation accelerating the dissolution and diffusion of oxygen; oxide formation; oxygen barrier effect of recrystallization and sintering microstructure in outer oxide scale. The alloying elements with high valence state and high diffusion rate in α-Ti are favorable to slow down the oxidation rate at the stage governed by oxide formation.

## 1. Introduction

Compared with nickel-based superalloys, high temperature near-α titanium alloys have advantages in specific strength, low-cycle fatigue property and fatigue crack propagation resistance in the range of 500~600 °C, based on which, new advanced aeroengines are in urgent need of the near-α titanium alloys for the purpose of weight reduction and thrust-weight ratio increase [1,2,3,4]. The typical near-α titanium alloys in the world are IMI834 from UK, Ti-1100 from USA, BT36 and BT41 from Russia, among which IMI834 has been successfully applied in EJ200, TRENT series, PW305 and PW150 engines [2]. TA29 titanium alloy is a near-α titanium alloy developed by China for the service characteristics and performance requirements of the advanced aeroengines with high thrust-weight ratio. Owing to its excellent thermal strength, good fracture toughness, plasticity and thermal stability, TA29 titanium alloy could be applied in disks, blades, blisks and casings of high-pressure compressors under long-term service temperature up to 600 °C, indicating that TA29 alloy is a new high-temperature titanium alloy with good application potential in aeroengines [3,4].

High temperature oxidation is one of the important issues impeding the application and development of high temperature titanium alloys in aeroengines [1,2,5,6]. At present, the research on high temperature oxidation of titanium alloys is mostly concerned with their long-term isothermal oxidation behavior at service temperature or slightly higher than service temperature [7,8,9,10]. However, in addition to isothermal oxidation, titanium alloys are often subject to non-isothermal oxidation during processing and service. For example, titanium alloy components in aeroengines suffer from non-isothermal oxidation under the induction of external energy (such as high-energy friction, fracture, melt droplets, etc.) and the produced heat leads to ignition and propagation combustion of titanium alloy components, which is the so-called titanium fire [11,12]. Therefore, the non-isothermal oxidation behavior of titanium alloys has a great influence on its safe application in aeroengines. Compared with the conventional isothermal oxidation, the non-isothermal oxidation of titanium alloys undergoes a shorter reaction time and a wider temperature range, of which the highest temperature is often higher than the normal service temperature (up to 600 °C) and even the lattice transition temperature (T_β_). Hence, the non-isothermal oxidation behavior of titanium alloys is more special than isothermal oxidation behavior.

The non-isothermal oxidation behavior of titanium alloy powders in micron- and nano-scales has been studied for the application in the fields of explosives and propellants [13,14], while the non-isothermal oxidation behavior of bulk titanium alloys is seldom studied. G.B. Mi et al. [15] studied the effect of Cr content on the non-isothermal oxidation behavior of Ti-Cr bulk alloy. The results showed that when the Cr content exceeds 10~15%, the oxidation resistance of Ti-Cr alloy increases with the Cr content due to the precipitation of Cr oxide.

In this paper, the non-isothermal oxidation behavior of TA29 titanium alloy was investigated by TGA/DSC method. Combined with microstructure characterization of non-isothermal oxidation products and calculation of oxidation activation energy, the influence mechanism of lattice transformation and alloying elements on the non-isothermal oxidation behavior was discussed and the non-isothermal oxidation mechanism of TA29 titanium alloy was revealed, which might provide guidance for improving the non-isothermal oxidation performance of high temperature titanium alloys.

## 2. Experimental

### 2.1. Non-Isothermal Oxidation Experiment

**Figure 1:**
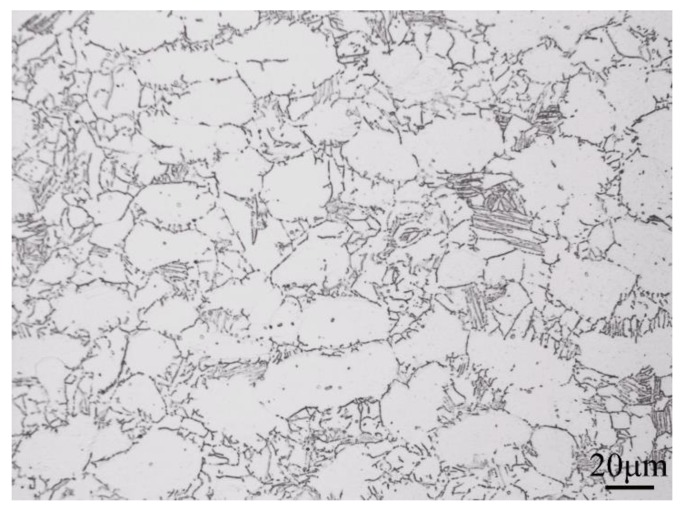
The initial microstructure of TA29 titanium alloy.

TA29 titanium alloy sheet with nominal composition of Ti-5.8Al-4Sn-4Zr-0.7Nb-1.5Ta-0.4Si-0.06C was obtained by smelting, forging, heat treatment and machining, the microstructures of which is shown in Figure 1. An α + β titanium alloy TC11 with nominal composition of Ti-6.5Al-3.5Mo-1.5Zr-0.3Si was selected for comparison of non-isothermal oxidation behavior. The oxide films on the titanium alloy sheets were polished with 600~2000 mesh sandpaper. Then specimens with size of 3 × 2 × 2 mm^3^ were cut by a computerized numerical control (CNC) dicing saw (SYJ-400, Shenyang kejing instrument company, Shenyang, China) followed by washing with acetone and alcohol. Non-isothermal oxidation experiments were carried out using TGA/DSC simultaneous thermal analyzer (TGA/DSC 1, Mettler Toledo, Zurich, Switzerland). The specimens were heated from room temperature to 1450 °C at a heating rate of 40 °C/min under a pure oxygen flow of 50 mL/min, and then cooled to room temperature and taken out of the furnace. Mass-gain curves were obtained from three repeated experiments and the relative standard deviations were less than 10%.

### 2.2. Microstructural Characterization

The non-isothermal oxidation products of TA29 titanium alloy were characterized. The phases and surface morphologies of the oxide scales were characterized by X-ray diffraction (XRD, D8 Advance, Cu Ka, Bruker, Karlsruhe, Germany) and field emission scanning electron microscopy (FE-SEM; SU8000, Hitachi, Toyko, Japan), respectively. To obtain metallographic specimens for cross-sectional morphology observation, the oxidized specimens were embedded in a cold-setting resin, then grinded with 400~2000 grit SiC sandpaper, polished with 1.0 μm alumina suspension, finally etched by Kroll reagent with 92 mL H_2_O, 3 mL HF and 5 mL HNO_3_. The microstructure of the substrate was observed with an optical microscope (OM, Zeiss upright microscopy Axio Lab A1, Zeiss, Oberkochen, Germany). The cross-sectional morphology and elemental distribution of the oxide scales were characterized by an electron probe microanalyzer (EPMA, Shimadzu EPMA-1720H, Shimadzu, Kyoto, Japan).

## 3. Calculation of Oxidation Activation Energy

Due to the high oxygen solubility of titanium alloys, the oxidation mass gain comes from oxygen dissolution in the alloy and oxide generation. However, the two oxidation behaviors are competitive and the mass gain is governed by one of them at a certain temperature and time range [16,17]. The rate-determining steps of the two oxidation behaviors are the diffusion of O atom in the alloy and the diffusion of O^2−^ in the oxide scale, respectively. Thus, it can be assumed that the mass-gain rate of titanium alloys during non-isothermal oxidation process is limited by one-dimensional diffusion of one specie A (A is O atom or O^2−^), so that the mass-gain rate per unit area of titanium alloys, $\Delta \dot{m}$, is proportional to the molar diffusion flux of specie A, *N*_A_:

(1) $$\Delta \dot{m}=kN_{A},$$

where the coefficient *k* is related to the stoichiometry of the oxidation reaction, the initial specimen mass and the type of specie A [18].

According to Fick’s diffusion law, the molar diffusion flux of specie A is expressed by

(2) $$N_{A}=D_{A}\frac{\Delta C_{A}}{\Delta z},$$

where *D*_A_ is the rate of specie A diffusing from the gas-solid interface to the reaction interface; ∆*z* and ∆*C*_A_ are the distance and the molar concentration difference of specie A between the gas-solid interface and the reaction interface, respectively. For the oxidation process governed by oxygen dissolution, the reaction interface refers to the interface of oxygen solid solution layer in the alloy, while for the oxidation process governed by oxide formation, the reaction interface refers to the oxide-substrate interface. In general, the concentration of specie A at the reaction interface is very low, so ∆*C*_A_ can be replaced by the concentration at the gas-solid interface, *C*_A_, and Equation (2) can be written as

(3) $$N_{A}=D_{A}\frac{C_{A}}{\Delta z},$$

From Equations (1) and (3), we can obtain the relationship between the mass gain per unit area of titanium alloys (∆*m*) and temperature:

(4) $$\frac{d(\Delta m)}{dT}=\frac{\Delta \dot{m}}{\dot{T}}=\frac{kN_{A}}{\beta }=\frac{kC_{A}D_{A}}{\beta \Delta z}=\frac{kC_{A}D_{A}}{\beta }\frac{\rho }{\Delta m},$$

where *β* is heating rate during non-isothermal oxidation process, *ρ* is the density of the region between the gas-solid interface and the reaction interface. For the oxidation process dominated by oxygen dissolution, *ρ* refers to the density of oxygen solid solution layer; while for the oxidation process dominated by oxide formation, *ρ* refers to the density of oxide scale.

Assuming that the diffusion of specie A satisfies Arrhenius kinetics, then

(5) $$D_{A}=A\exp(-E/RT),$$

where A is the pre-exponential factor, *E* is the oxidation activation energy and R is the molar gas constant. From Equations (4) and (5), we obtain

(6) $$\frac{d(\Delta m)}{dT}=K^{*}\frac{\exp(-E/RT)}{\Delta m},$$

where $K^{*}=kC_{A}A\rho /\beta$, which is constant at certain temperature and time range.

Take logarithms on both sides of Equation (6) and rewrite it,

(7) $$-\ln\frac{d(\Delta m)}{dT}-\ln(\Delta m)=-\ln K^{*}+E/RT,$$

let the left side of Equation (7) equal to *Y*(∆*m*), thus *Y*(∆*m*) depends linearly on the reciprocal of temperature. The positive slope of the fitted line is multiplied by R to get the oxidation activation energy.

## 4. Results

### 4.1. Non-Isothermal Oxidation Mass-Gain Curve

**Figure 2:**
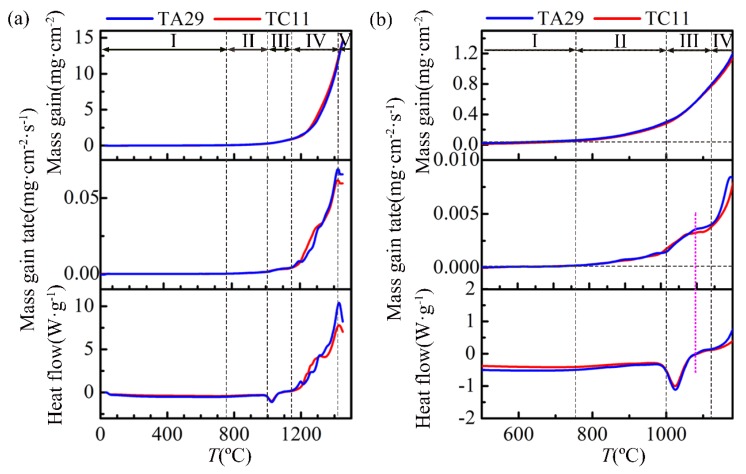
The mass-gain curves, mass-gain rate curves and heat flux curves of TA29 and TC11 titanium alloys during non-isothermal oxidation process: (**a**) 30~1450 °C; (**b**) the local enlarged diagram of Figure 2a.

The mass-gain curve, mass-gain rate curve and heat flow curve of TA29 titanium alloy during non-isothermal oxidation are shown in Figure 2, among which Figure 2b is a local enlarged diagram of Figure 2a. According to the variation of the mass-gain rate, the non-isothermal oxidation process of TA29 titanium alloy is divided into five stages. When the temperature is lower than 750 °C (Stage I), the mass gain can be neglected. When the temperature rises to 750~1000 °C (Stage II), the mass gain rate increases slowly and the mass gain of this stage is 0.2 mg∙cm^−2^. When the temperature is increased to 1000~1120 °C (Stage III), the mass gain rate obviously increases and then tends to be steady, leading to the mass gain of 0.5 mg∙cm^−2^. There is a concave endothermic peak in the heat flow curve at this stage which might be related to phase transition of the titanium alloy or its oxidation products. The termination temperature of the endothermic peak corresponds to the critical temperature where the mass gain rate tends to be steady, as shown by the dotted lavender line in Figure 2b, indicating that the obvious increase of the mass gain rate is related to the phase transition corresponding to the endothermic peak. When the temperature reaches 1120~1420 °C (Stage IV), the mass gain rate sharply increases and the mass gain is 11.5 mg∙cm^−2^. However, when the temperature exceeds 1420 °C (Stage V), the mass gain rate and oxidation heat volume decreases and the mass gain is 2.4 mg∙cm^−2^.

**Figure 3:**
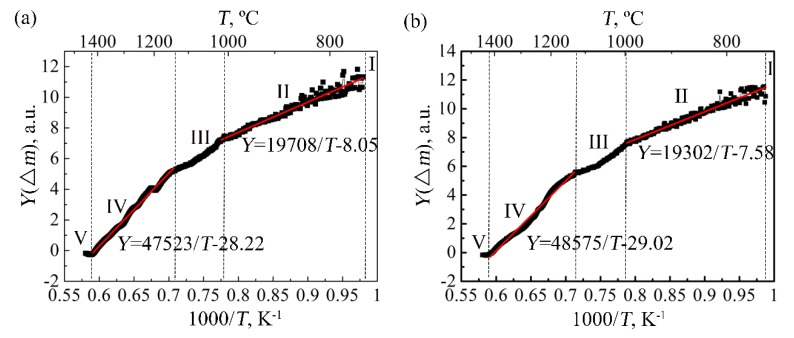
The relationship curves between Y(∆m) and 1/*T* and the corresponding linear fitting results for the titanium alloys: (**a**) TA29; (**b**) TC11.

The relationship curve between *Y*(∆*m*) and 1/*T* and the corresponding linear fitting results for TA29 alloy are shown in Figure 3a. The curve also indicates that there are five stages in the non-isothermal oxidation process. Since the mass change at Stage I mainly comes from the mass fluctuation of the thermogravimetric analysis equipment, the values of *Y*(∆*m*) at this stage fluctuate around a certain value and are not shown in Figure 3a. Both for Stage II and Stage IV, the relationship curves between *Y*(∆*m*) and 1/*T* could be positive linear fitted and the oxidation activation energies of the two stages are obtained, which is 163.9 kJ∙mol^−1^ and 395.1 kJ∙mol^−1^, respectively. While for Stage III and Stage V, the relationship curve between *Y*(∆*m*) and 1/*T* could not be positively linear fitted, indicating that the oxidation mechanisms of the two stages is somewhat different from those of Stage II and Stage IV.

Therefore, there are five oxidation stages in the non-isothermal oxidation process of TA29 titanium alloy, including no oxidation (Stage I, <750 °C), slow oxidation (Stage II, 750~1000 °C), accelerated oxidation (Stage III, 1000~1120 °C), severe oxidation (Stage IV, 1120~1420 °C) and deceleration oxidation (Stage V, 1420~1450 °C). Similarly, the non-isothermal oxidation process of the α + β titanium alloy TC11 can be also divided into the five oxidation stages, as shown in Figure 2 and Figure 3b. It should be noted that the experimental conditions of non-isothermal oxidation such as heating rate and ambient atmosphere may cause some deviation in the temperature ranges of the oxidation stages. Therefore, the temperature ranges for the oxidation stages involved in this paper refer to the result under specific experimental conditions.

### 4.2. Morphology of Oxide Scale

**Figure 4:**
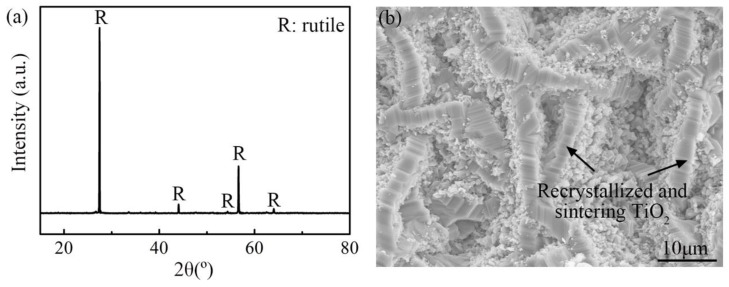
(**a**)The XRD pattern and (**b**) the surface morphology of the oxide scale on TA29 alloy.

The XRD pattern with sharp diffraction peaks in Figure 4a shows that the oxidation product detected is rutile with high crystallinity. In addition, the surface morphology of the oxide scale is composed of coarse strip TiO_2_ crystals, as shown in Figure 4b, which is a typical recrystallization and sintering structure. Both the XRD pattern and the surface morphology manifest the occurrence of recrystallization and sintering of the oxide scale during non-isothermal oxidation process.

**Figure 5:**
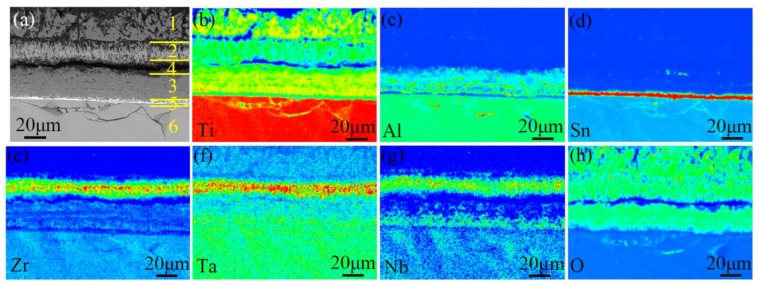
(**a**) The cross-sectional morphology and (**b**–**h**) the corresponding EPMA mapping results of the oxide scale on TA29 alloy.

The cross-sectional morphology of the oxide scale and the corresponding elemental distribution by EPMA mapping are shown in Figure 5. The oxide scale consists of three layers (marked as 1~3 in Figure 5a): the outer layer with coarse TiO_2_ grain structure, intermediate layer with Zr, Nb, Ta-rich TiO_2_ structure and inner layer with fine Al-rich TiO_2_ structure, the thicknesses of which are about ~28 μm, ~19 μm and ~27 μm, respectively. There is a transverse crack between the intermediate layer and the inner layer (marked as 4 in Figure 5a). A thin Sn-rich titanium layer is formed at the oxide-substrate interface (marked as 5 in Figure 5a), below which there is a hard brittle layer of oxygen-stabilized a-case (marked as 6 in Figure 5a). It shows that the alloying elements distribute in different positions of the oxide scale on TA29 titanium alloy. There is no alloying element doping in the outer oxide layer, indicating that this layer is formed by outward growth through the diffusion of Ti^4+^ from the titanium alloy substrate to the oxide-gas interface, while there is exit alloying elements doping in the intermediate and inner oxide layers, indicating that the two layers are generated by inward growth through the diffusion of O^2−^ from the oxide-gas interface to the titanium alloy substrate. The thickness of the inward-growth oxide accounts for about 62% of the total thickness, which is similar to that of TiAl-based alloy (70%) [19]. It demonstrates that the oxide growth on TA29 titanium alloy is dominated by the inward diffusion of O^2−^.

### 4.3. Morphology of Substrate

**Figure 6:**
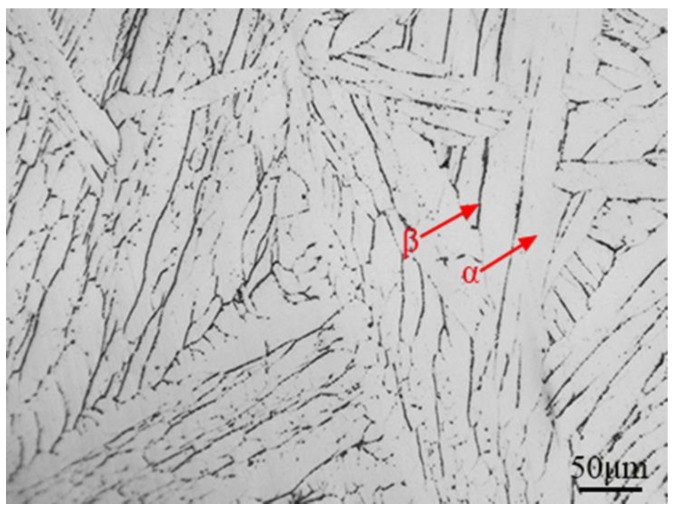
The microstructure of the TA29 alloy substrate after non-isothermal oxidation.

Figure 6 shows the substrate structure of TA29 titanium alloy after non-isothermal oxidation experiment. It shows β phase as a small seam around the coarse α lamellar, which is similar to the typical structure of titanium alloys formed through nucleation of α phase at the β grain boundary and subsequent layered-pattern growth of the α phase into the β grain when temperature drops below β-transition temperature (T_β_) [1]. The substrate microstructure indicates that TA29 titanium alloy undergoes the phase transition of α→β during non-isothermal oxidation process. In addition, there is an endothermic peak around 1025 °C in the heat flux curve during non-isothermal oxidation (Figure 2b), which may correspond to phase transition of the titanium alloy or its oxidation products. Generally, the oxidation product of titanium alloys is only rutile under atmospheric pressure [20]. Even if there are some other phases of TiO_2_ in the oxidation products, the phase transitions of TiO_2_ during heating process involve anatase→rutile (700 °C, endothermic) and brookite→rutile (900 °C, exothermic) [21], which does not match the endothermic peak in Figure 2b. Thus, the endothermic peak may only correspond to the phase transformation of the titanium alloy instead of the oxidation products. Besides melting, the phase transition of TA29 titanium alloy only involves α→β during heating process from the Ti-Al phase diagram [22] and the transition temperature (T_β_) is 1050 °C [23], which is similar to the peak temperature of the endothermic peak (1025 °C). Therefore, the titanium alloy undergoes the phase transition α→β corresponding to the endothermic peak in Figure 2b.

## 5. Discussion

Based on the aforementioned mass-gain curve, oxidation activation energies and morphologies of oxide scale and substrate, the non-isothermal oxidation mechanisms at different stages are revealed and the influence of lattice transformation and alloying elements on the oxidation behavior of TA29 alloy is clarified.

### 5.1. Stage I

The mass gain of TA29 titanium alloy at Stage I (<750 °C) can be neglected (Figure 2b), the reason for which is that the diffusion rate of oxygen is small at low temperature and a thin compact TiO_2_ film formed on the titanium alloy could act as an oxygen barrier.

### 5.2. Stage II

When the temperature rises to Stage II (750~1000 °C), the diffusion rate of oxygen increases and the TA29 titanium alloy enters the slow oxidation stage. Kofstad [17] found that more than 80% of the reacted oxygen had dissolved in titanium after oxidation at 900 °C for 30 min, indicating that the oxygen dissolution in titanium was the main source of mass gain under such oxidation condition. In this work, the time required for heating from 750 °C to 900 °C and from 900 °C to 1000 °C were 3.75 min and 2.5 min, respectively. Since the parabolic rate constants for titanium oxidation governed by oxygen dissolution at 900 °C and 1000 °C are 0.057 mg·cm^−2^·min^−1^ and 0.406 mg·cm^−2^·min^−1^, respectively [17], the non-isothermal oxidation from 900 °C to 1000 °C is approximately equivalent to oxidation at 900 °C for 17.8 min. If the differences of oxygen solubility and oxygen diffusion coefficient between TA29 alloy and pure titanium could be neglected, the non-isothermal oxidation of TA29 titanium alloy at Stage II is equivalent to oxidation at 900 °C for 21.6 min. Thus, the oxidation behavior of TA29 titanium alloy at this stage should be similar to that of titanium oxidized at 900 °C for 30 min, namely, the oxygen dissolution is the main source of oxidation mass gain. Furthermore, the oxidation activation energy of TA29 titanium alloy at Stage II is 163.9 kJ∙mol^−1^, which is in agreement with the diffusion activation energies of oxygen in α-Ti and β-Ti at this temperature range (140~250 kJ∙mol^−1^ and 130~288 kJ∙mol^−1^, respectively) [24]. It further indicates that the mass gain source at this stage is oxygen dissolution with the rate-determining step of oxygen diffusion in the alloy.

Since the microstructure of TA29 alloy at Stage II is mainly α phase, the oxidation rate depends on the diffusion rate of oxygen in the α phase. However, the alloying elements might affect the oxygen diffusion coefficient through interacting with interstitial O atoms in the α-Ti lattice. The alloying elements of TA29 alloy mainly include Al, Sn, Zr, Nb and Ta, existing in the α-Ti lattice as displaced solute atoms. Taking the interaction force between Ti and O in α-Ti lattice as the zero reference, the interaction between Zr and O is very weak since both Zr and Ti are Group IV-B elements, thus Zr has little effect on the oxygen diffusion coefficient in α-Ti lattice [25]. Due to the slightly higher d-orbital filling of Nb and Ta than that of Ti (The electron configurations are 4p^6^4d^4^5s^1^, 5d^3^6s^2^ and 3p^6^3d^2^4s^2^ for Nb, Ta and Ti, respectively), Nb and Ta slightly reduce the oxygen diffusion coefficient in α-Ti lattice [25]. Al and Sn tend to hinder oxygen diffusion through repelling or destabilizing the interstitial O atoms, while the hindering effect is not significant due to the low contents of Al and Sn [25]. Accordingly, Zr has little effect on the oxidation behavior, while Nb, Ta, Al, Sn modestly reduce the oxidation rate at Stage II governed by oxygen dissolution.

### 5.3. Stage III

When the temperature reaches Stage III (1000–1120 °C), the mass-gain rate obviously increases and then tends to stabilize, TA29 alloy enters the accelerated oxidation stage. As mentioned in Section 4, the obvious increase of the mass-gain rate is related to the phase transition of α→β. Therefore, the effect of lattice structure on the oxygen diffusion coefficient and oxygen solubility in α and β phases is discussed firstly, followed by the effect of the lattice transition on the oxidation behavior of the titanium alloy. 

**Figure 7:**
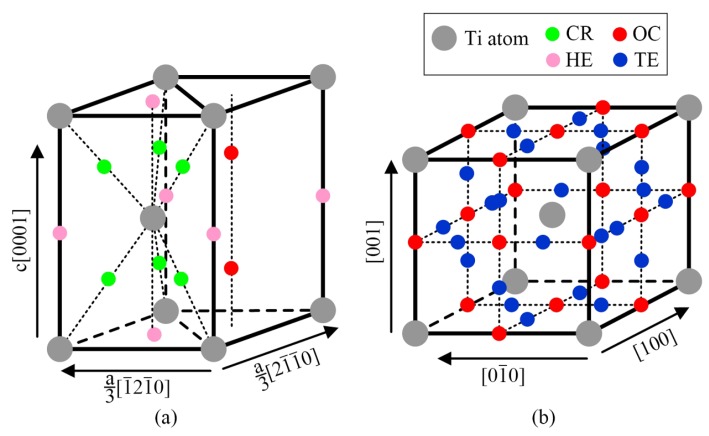
The stable interstitial sites for O atoms in (**a**) α-Ti lattice and (**b**) β-Ti lattice. Crowdion (CR), hexahedral (HE), octahedral (OC) and tetrahedral (TE) represent crowdion, hexahedral, octahedral and tetrahedral interstitial sites, respectively.

α-Ti is a close-packed hexagonal crystal structure (hcp, A3), where there exit eight kinds of interstitial sites: octahedral (OC), tetrahedral (TE), crowdion (CR), split dumbbells along the c axis (SP), basal octahedral (BO), hexahedral (HE), basal crowdion (BC) and split dumbbells in the basal plane (BS) [26]. Among these interstitial sites, only three interstitial sites, namely, OC, CR and HE as shown in Figure 7a, are stable for O atoms and the OC interstitial site is most stable [26,27]. However, the energy barrier and migration frequency for oxygen diffusion in α-Ti with different transition pathways (Table 1 [26,27]) show that it is difficult for O atoms to jump directly from one OC interstitial site to another one, which could be more easily implemented with the aid of other interstitial sites, such as OC→CR→OC with the energy barrier of about 2.5 eV. β-Ti is a body-centered cubic crystal structure (bcc, A2), in which there exist octahedral (OC) and tetrahedral interstitial sites (TE), as shown in Figure 7b. Similarly, O atoms prefer to occupy the OC interstitial site in body-centered cubic metals [28,29] and the migration of O atoms from one OC interstitial site to another one is more easily achieved with the help of other interstitial site, such as OC→TE→OC with the energy barrier of about 1.23 eV [28].

**Figure 8:**
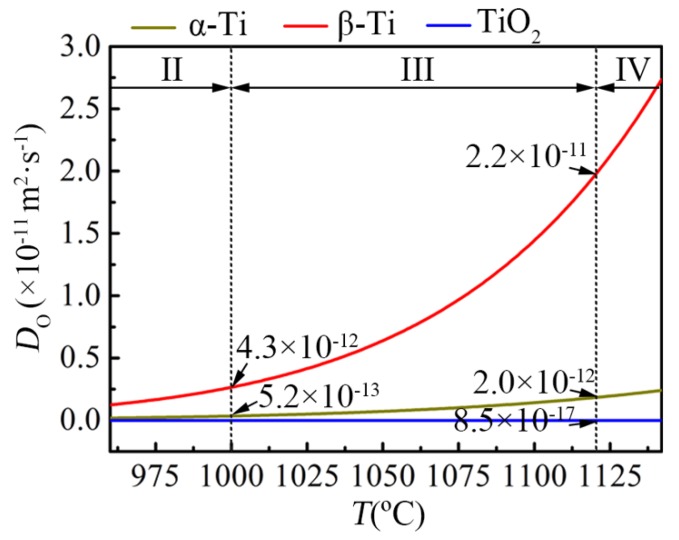
The comparison of oxygen diffusion coefficients in α-Ti, β-Ti and TiO_2_. The diffusion coefficients of O atom in α-Ti and β-Ti are respectively expressed by $D_{\alpha }(m^{2}\cdot s^{-1})=7.78\times 10^{-5}\exp(-203400/RT)$ and $D_{\beta }(m^{2}\cdot s^{-1})=3.3\times 10^{-2}\exp(-246000/RT)$ [30]. The diffusion coefficient of O^2−^ in TiO_2_ is expressed by $D_{{\mathrm{TiO}}_{2}}(m^{2}\cdot s^{-1})=1.7\times 10^{-6}\exp(-276000/RT)$ [24].

It can be seen that the energy barrier for oxygen diffusion in bcc lattice is smaller than that in hcp lattice. Hence, the oxygen diffusion coefficient in β-Ti is one order of magnitude higher than that in α-Ti, as shown in Figure 8. Besides, O atoms tend to occupy octahedral interstitial sites in both α-Ti and β-Ti. The octahedral interstice in the α-Ti lattice is symmetrical with the size of 54.6 pm (0.414 *r*_Ti_), while the octahedral interstice in the β-Ti lattice is asymmetrical with the sizes of 20.3 pm (0.154 *r*_Ti_) and 83.6 pm (0.633 *r*_Ti_) along the <100> and <110> directions, respectively. Though the size along the <110> direction of the octahedral interstice in the β-Ti lattice is much larger than oxygen atom radius (66 pm), the oxygen dissolution in the β-Ti lattice requires more energy to push away the two Ti atoms in the <100> direction compared to that in the α-Ti lattice. Hence, the oxygen solubility of β-Ti is much smaller than that of α-Ti (0.8~3.8 at.% and 34 at.%, respectively) [30].

**Figure 9:**
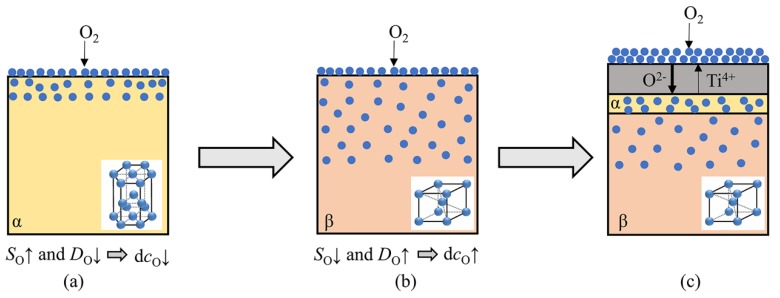
Schematic diagram of oxygen distribution in TA29 titanium alloy at (**a**) Stage II; (**b**) Stage III and (**c**) Stage IV. *S*_O_ and *D*_O_ represent the solubility and diffusion coefficient of oxygen in the alloy, respectively; d*c*_O_ represents the oxygen concentration gradient between the alloy surface and the oxygen solid solution layer.

Based on the differences of oxygen solubility and oxygen diffusion coefficient in α-Ti and β-Ti, the accelerated oxidation behavior related to the lattice transition of α→β could be explained as follows. Before the occurrence of the lattice transition (Stage II), the titanium alloy substrate is dominated by α phase. Due to the high oxygen solubility and low oxygen diffusion coefficient of α-Ti, oxygen atoms greatly dissolute and gather at the subsurface of the alloy, leading to a small oxygen concentration gradient between the alloy surface and the oxygen solid solution layer. As a result, the diffusion driving force of O atoms from the alloy surface into the bulk is low and the oxidation mass-gain rate is small, as shown in Figure 9a. However, when the lattice transition of α→β occurs (Stage III), the great increase of the oxygen diffusion coefficient causes the rapid diffusion of O atoms and the formation of a thicker oxygen solid solution layer. Together with the remarkable decrease of the oxygen solubility, the oxygen concentration gradient significantly increases between the alloy surface and the oxygen solid solution layer, resulting in a marked increase of the oxygen diffusion driving force and the oxidation mass-gain rate, as shown in Figure 9b. When the lattice transition is over, the oxidation rate tends to stabilize since the oxygen solubility and oxygen diffusion coefficient in the alloy become constant. The above analysis manifests that the oxidation behavior at Stage III still belongs to the oxidation process dominated by oxygen dissolution with the rate-determining step of oxygen diffusion, but the changes in oxygen solubility and oxygen diffusion coefficient caused by lattice transition make the oxidation behavior at this stage slightly different from that at Stage II.

### 5.4. Stage IV

When the temperature raises to Stage IV (1120~1420 °C), the TA29 titanium alloy enters the severe oxidation stage. Generally, oxidation of titanium alloys involves oxygen dissolution and oxide formation. When the oxygen content in the subsurface of titanium alloys reaches the solubility limit, the nucleus of TiO_2_ will precipitate and grow into a film [17]. Furthermore, the oxidation activation energy of TA29 titanium alloy at this stage is 395.1 kJ∙mol^−1^, which is closer to the activation energy of oxygen diffusion in TiO_2_ at this temperature range (276 kJ∙mol^−1^) [24] compared to that of oxygen diffusion in titanium. Therefore, the main oxidation mechanism of TA29 titanium alloy at this stage is the formation of TiO_2_ scale. Besides the oxide formation, Stage IV involves the phase transition from oxygen-saturated β phase in the subsurface to oxygen-stabilized α case [20] since oxygen is an α-stabilizing element. The occurrence of the phase transition could be further confirmed by the morphology of the oxygen-enriched α case in the subsurface of TA29 titanium alloy (Figure 5a), which is also a possible reason for the higher activation energy at this stage than that of oxygen diffusion in TiO_2_.

As mentioned in Section 4.2, the growth of the oxide scale on TA29 alloy is dominated by the inward diffusion of O^2−^. In addition, the diffusion coefficient of O^2−^ in TiO_2_ is several orders of magnitude lower than that of O atom in α-Ti and β-Ti, as shown in Figure 8. Hence, the oxidation mass-gain rate at Stage IV depends on the diffusion rate of O^2−^ in the TiO_2_ oxide scale, while the oxygen solubility and oxygen diffusion coefficient in the substrate have no obvious effect on the mass-gain rate, as shown in Figure 9c. That is to say, the lattice transition from oxygen-saturated β phase to oxygen-stabilized α case in the subsurface layer of the alloy has little effect on the oxidation behavior of TA29 alloy at the stage dominated by the oxide formation.

**Figure 10:**
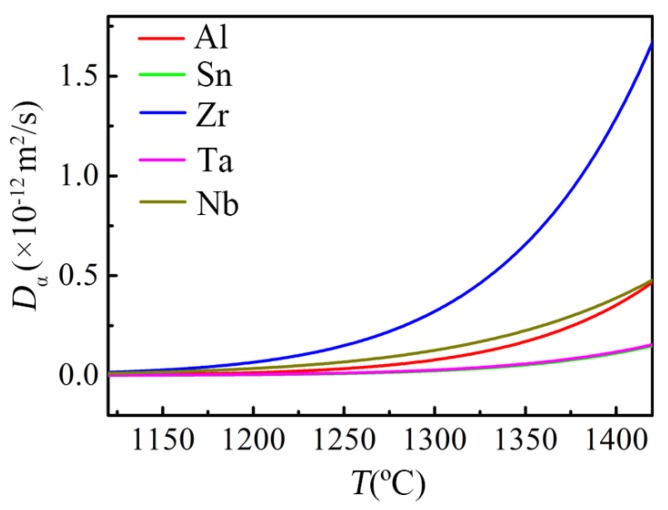
The diffusion coefficients of the alloying elements in α-Ti at the temperature range of Stage IV, which is obtained by extrapolating the expressions in Table 2 to higher temperature range.

The alloying elements distribute differently in the oxide scale, which is embodied in that Zr, Nb and Ta are enriched in the intermediate oxide layer, Al is enriched in the inner oxide layer, while Sn is segregated at the oxide-substrate interface (Figure 5). T. Kitashima et al. [31] considered that the segregation of Sn at the oxide-substrate interface is due to the low solubility of Sn in TiO_2_. However, it can be seen from the phase diagrams [32,33,34,35] that the solubility of Sn in TiO_2_ is higher than that of the other alloying elements at the temperature range of Stage IV, as shown in Table 2, indicating that the segregation of Sn at the oxide-substrate interface should be due to other factors. Actually, the distribution of the alloying elements might be related to their diffusion coefficients in the subsurface α case since the alloying elements are enriched in the inward-growth oxide layers and the oxide-substrate interface. The diffusion coefficients of the alloying elements in α-Ti at the temperature range of Stage IV are arranged in order: D(Zr) > D(Nb) > D(Al) > D(Ta) > D(Sn), as shown in Figure 10 and Table 2 [36,37], which is basically in accordance with the distribution of the alloying elements in the oxide scales except for Ta. That is, Zr and Nb with larger diffusion coefficients could diffuse rapidly and gather at the near-surface region during the growth of the intermediate oxide layer so as to realize their doping in the intermediate oxide layer. Similarly, Al with smaller diffusion coefficient diffuses relatively slow and dopes in the inner oxide layer, while Sn with the smallest diffusion coefficient is segregated at the oxide-substrate interface. It demonstrates that there is a correlation between the distribution of alloying elements in the oxide scale and the diffusion rate of alloying elements in the subsurface α case, but the specific mechanism still needs further study.

The distribution of the alloying elements in the oxide scale indicates that the alloying elements participate in the formation of the oxide scale and then affect the oxidation rate. The doping of Nb^5+^ and Ta^5+^ in the intermediate oxide layer decreases the oxygen vacancy concentration in the TiO_2_ lattice, which slows down the growth rate of the TiO_2_ scale. Thus, Nb and Ta elements play an important role in improving the oxidation resistance. However, the doping of Al^3+^ in the inner oxide layer increases the oxygen vacancy concentration in the TiO_2_ lattice, which promotes the growth of the TiO_2_ scale. Hence, a small amount of Al decreases the oxidation resistance of TA29 alloy. As for the segregation of Sn at the oxide-substrate interface, on the one hand, it increases the vacancy concentration in the alloy, promoting the diffusion of Ti atoms in the alloy; on the other hand, it reduces the adhesion between the oxide scale and the substrate, prompting the oxide scale to peel off [38,39,40]. Both intensify the reaction between Ti and O and decrease the oxidation resistance of the alloy, thus Sn is detrimental to the oxidation resistance of TA29 alloy. 

Based on the aforementioned discussion, it shows that the alloying elements with high valence state and high diffusion rate in α-Ti is favorable to decrease the oxidation rate in Stage IV.

### 5.5. Stage V

When the temperature reaches Stage V (1420~1450 °C), the mass-gain rate of TA29 titanium alloy decreases, indicating that the diffusion of the reacting species in the oxide scale is blocked. Kofstad [17] found that recrystallization and sintering occur in the outer TiO_2_ layer when temperature exceeds 950 °C, accompanied by the decrease of the oxidation rate. Furthermore, he found that the time required for the occurrence of recrystallization is greatly shortened with the increase of temperature. For example, the time required was 5 min at 1200 °C instead of 10~15 h at 950 °C [17]. In this work, the heating time from 1200 °C to 1420 °C was 5.5 min, thus the outer TiO_2_ layer has already met the recrystallization occurrence condition when the temperature reaches Stage V, which is also confirmed by the surface morphology and XRD pattern of the oxide scale (Figure 4). As a result, the reason for the decelerated oxidation behavior of TA29 alloy at Stage V is that the occurrence of recrystallization and sintering of the outer oxide layer, which hinders the diffusion of the reacting species.

**Figure 11:**
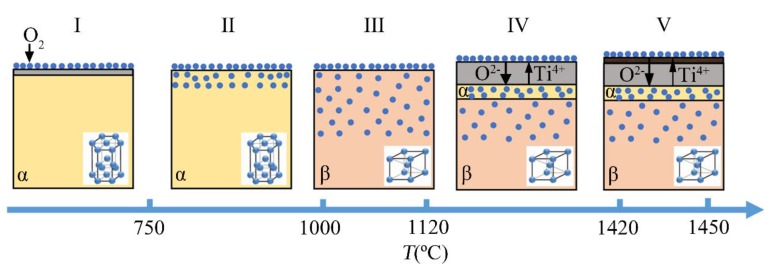
Schematic diagram of non-isothermal oxidation mechanism for TA29 titanium alloy.

In summary, the non-isothermal oxidation mechanism of the near-α titanium alloy TA29 heating from room temperature to 1450 °C at a heating rate of 40 °C/min in pure oxygen atmosphere can be summarized as follows (Figure 11): When the temperature is lower than 750 °C (Stage I), the negligible mass gain results from the low oxygen diffusion ability and a thin compact oxide film on the titanium alloy acting as an oxygen barrier; when the temperature rises to 750~1000 °C (Stage II), the slow oxidation behavior is due to the oxygen dissolution with the rate-determining step of oxygen diffusion in the alloy; when the temperature is raised to 1000~1120 °C (Stage III), the accelerated oxidation behavior is related to the lattice transformation of α→β, which promotes the dissolution and diffusion of oxygen in the alloy; when the temperature reaches 1000~1120 °C (Stage III), the severe oxidation behavior stems from the formation of TiO_2_ oxide scale; when the temperature exceeded 1420 °C (Stage V), the decelerated oxidation behavior is caused by the occurrence of recrystallization and sintering in the outer TiO_2_ layer.

## 6. Conclusions

1. The oxide scale on TA29 titanium alloy after non-isothermal oxidation is a three-layer structure of TiO_2_. The outer layer is coarse recrystallized and sintering structure without doping elements, while the intermediate layer is Zr, Nb, Ta-rich TiO_2_ structure, the inner layer is fine Al-rich TiO_2_ structure, and Sn is segregated at the oxide-substrate interface. The distribution of the alloying elements in the oxide scale is related to their diffusion rates in the subsurface α case.
2. The non-isothermal oxidation process of TA29 titanium alloy can be divided into five stages, including no oxidation, slow oxidation, accelerated oxidation, severe oxidation and decelerated oxidation, the oxidation mechanisms of which are as follows: Oxygen barrier effect of a thin compact oxide film on the titanium alloy; oxygen dissolution in the alloy; lattice transformation of α→β promoting the dissolution and diffusion of oxygen; formation of TiO_2_ scale; the occurrence of recrystallization and sintering in the outer oxide layer inhibiting the diffusion of reacting species.
3. In the oxidation stage dominated by oxygen dissolution, lattice transformation of the alloy obviously promotes the oxidation rate; as for the alloying elements, Zr has little effect on the oxidation rate, while Nb, Ta, Al and Sn modestly reduce the oxidation rate at this stage.
4. In the oxidation stage dominated by oxide formation, the lattice transformation in the subsurface layer of the alloy has little effect on the oxidation behavior; as for the alloying elements, Zr has little effect on the oxidation rate, Nb and Ta reduce the oxidation rate of the alloy, while a small amount of Al increase the oxidation rate and Sn deteriorates the oxidation resistance.

## Figures and Tables

**Table 1:** The energy barrier and migration frequency for oxygen diffusion in α-Ti lattice with different transition pathways.

Transition Pathway	Energy Barrier (eV)		Migration Frequency (THz)	
	Ref. [26]	Ref. [27]	Ref. [26]	Ref. [27]
OC→OC	3.85	3.25	13.67	11.76
OC→HE	2.061	2.04	12.24	10.33
OC→CR	1.883	2.16	27.92	16.84
HE→OC	0.833	0.85	10.45	5.58
HE→CR	0.676	0.94	5.7	10.27
CR→OC	0.575	0.28	11.19	12.21
CR→HE	0.596	0.24	3.6	13.81

**Table 2:** Solubility of the alloying elements in TiO_2_ and diffusion coefficient of the alloying elements in α-Ti.

Alloying Element	Solubility in TiO_2_ at 1120~1420 °C (at.%)	Diffusion in α-Ti at 600~827 °C [36] (m^2^∙s^−1^)
Al	0.2~0.4 [32]	6.6 × 10^−3^exp(−329000/RT)
Sn	3.6~12.2 [33]	4 × 10^−3^exp(−338000/RT)
Zr	1.0~3.0 [34]	4 × 10^−3^exp(−304000/RT)
Nb	4.6~5.9 [35]	1.835 × 10^−5^exp(−29565/T) [37]
Ta	Unknown	1 × 10^−3^exp(−318000/RT)
